# Ocular Application of the Kinin B_1_ Receptor Antagonist LF22-0542 Inhibits Retinal Inflammation and Oxidative Stress in Streptozotocin-Diabetic Rats

**DOI:** 10.1371/journal.pone.0033864

**Published:** 2012-03-28

**Authors:** Mylène Pouliot, Sébastien Talbot, Jacques Sénécal, Florence Dotigny, Elvire Vaucher, Réjean Couture

**Affiliations:** 1 École d'optométrie, Université de Montréal, Montréal, Canada; 2 Département de Physiologie, Faculté de Médecine, Université de Montréal, Montréal, Canada; University of Florida, United States of America

## Abstract

**Purpose:**

Kinin B_1_ receptor (B_1_R) is upregulated in retina of Streptozotocin (STZ)-diabetic rats and contributes to vasodilation of retinal microvessels and breakdown of the blood-retinal barrier. Systemic treatment with B_1_R antagonists reversed the increased retinal plasma extravasation in STZ rats. The present study aims at determining whether ocular application of a water soluble B_1_R antagonist could reverse diabetes-induced retinal inflammation and oxidative stress.

**Methods:**

Wistar rats were made diabetic with STZ (65 mg/kg, i.p.) and 7 days later, they received one eye drop application of LF22-0542 (1% in saline) twice a day for a 7 day-period. The impact was determined on retinal vascular permeability (Evans blue exudation), leukostasis (leukocyte infiltration using Fluorescein-isothiocyanate (FITC)-coupled Concanavalin A lectin), retinal mRNA levels (by qRT-PCR) of inflammatory (B_1_R, iNOS, COX-2, ICAM-1, VEGF-A, VEGF receptor type 2, IL-1β and HIF-1α) and anti-inflammatory (B_2_R, eNOS) markers and retinal level of superoxide anion (dihydroethidium staining).

**Results:**

Retinal plasma extravasation, leukostasis and mRNA levels of B_1_R, iNOS, COX-2, VEGF receptor type 2, IL-1β and HIF-1α were significantly increased in diabetic retinae compared to control rats. All these abnormalities were reversed to control values in diabetic rats treated with LF22-0542. B_1_R antagonist also significantly inhibited the increased production of superoxide anion in diabetic retinae.

**Conclusion:**

B_1_R displays a pathological role in the early stage of diabetes by increasing oxidative stress and pro-inflammatory mediators involved in retinal vascular alterations. Hence, topical application of kinin B_1_R antagonist appears a highly promising novel approach for the treatment of diabetic retinopathy.

## Introduction

Recent findings suggest a role for the kallikrein-kinin system in the development of diabetic retinopathy [1], [2], [3]. Kinins are important inflammatory mediators involved in tissue edema, leukocytes infiltration, vasodilation and regulation of local blood flow [4]. These peptides are produced at the site of inflammation and exert their effects through the activation of two G-protein-coupled receptors named B_1_ (B_1_R) and B_2_ (B_2_R) [5], [6]. B_2_R is constitutively expressed and mediates mainly the acute effects of kinins due to its rapid desensitization. In contrast, B_1_R is expressed at very low levels in physiological conditions. This inducible receptor is upregulated in response to tissue injury, by pro-inflammatory cytokines or by the oxidative stress associated to hyperglycemia. The B_1_R is involved in the chronic phase of the inflammatory response which is compatible with its low desensitization mechanism [7], [8], [9]. Bradykinin (BK) and kallidin (KD) mediate the action of B_2_R while their kininase I metabolites des-Arg^9^-BK and des-Arg^10^-KD are the preferential agonists for B_1_R [7], [8]. The activation of these receptors induces the release of nitric oxide (NO), prostaglandins and pro-inflammatory cytokines [4], [10], [11].

Most components of the kallikrein-kinin system have been identified in the human, rabbit and rat retina [12], [13], [14], [15]. Particularly, B_1_R was found overexpressed in the retina of Streptozotocin (STZ)-diabetic rats through a mechanism involving oxidative stress [16], [17]. In STZ-diabetic rats, B_1_R mediates vasodilation of *ex vivo* retinal microvessels [16] and contributes to the breakdown of the blood-retinal barrier *in vivo* [17]. Collectively, these results suggest a pathological role for B_1_R in the development of retinal damage in diabetes and the progression of diabetic retinopathy.

Diabetic retinopathy is characterized by vascular alterations including retinal blood flow changes, endothelial cells dysfunction, breakdown of the blood-retinal barrier, ischemia and neovascularisation [18]. Oxidative stress and inflammatory processes are thought to contribute largely to the development of the disease [19], [20], [21]. Hyperglycemia induces the production of reactive oxygen species (ROS) and the expression of many pro-inflammatory factors in the diabetic retina including inducible nitric oxide synthase (iNOS), interleukin-1β (IL-1β), cyclooxygenase-2 (COX-2) and vascular endothelial growth factor (VEGF) [20]. Vasodilation, increased vascular permeability and adhesion of inflammatory cells to the vascular wall are also part of the inflammatory response occurring in the retina during diabetes [22], [23], [24].

The present study aims at determining whether topical ocular application of LF22-0542, a non-peptide water soluble B_1_R antagonist, could reverse diabetes-induced retinal inflammation and oxidative stress. Our data show that the enhanced retinal vascular permeability, leukostasis, the enhanced expression of several inflammatory mediators and higher production of superoxide anion levels were significantly decreased in the retina of STZ-diabetic rats treated with LF22-0542. This provides the first demonstration that ocular application of a kinin B_1_R antagonist could be an effective strategy in the treatment of diabetic retinopathy.

## Materials and Methods

### STZ-diabetic rats

All experimental methods and animal care procedures were approved by the animal care committee of the Université de Montréal (protocol 09-030), in accordance with the Canadian Council on Animal Care. Male Wistar rats weighting 200–250 g were purchased from Charles River (St-Constant, QC, Canada) and housed two per cage in a room under controlled temperature (23°C), humidity (50%) and lighting (12-hour light/dark cycle) with food and water provided *ad libitum*. Rats were rendered diabetic by a single i.p. injection of Streptozotocin (STZ, Zanosar 65 mg/kg, Sigma-Aldrich, Oakville, ON, Canada). Age-matched control rats were injected with vehicle (sterile saline 0.9%, pH. 7.4). Glucose concentrations were measured in blood samples obtained from the tail vein with a commercial blood glucose analyzer (Accusoft; Roche Diagnostics, Laval, QC, Canada). Only STZ-treated rats with blood glucose concentration higher than 20 mmol/L were considered as diabetic and included in the study. Glycemia and body weight were recorded twice a week and on the day of the experiment.

### Topical ocular treatment with B_1_R antagonist LF22-0542

**Figure 1:**
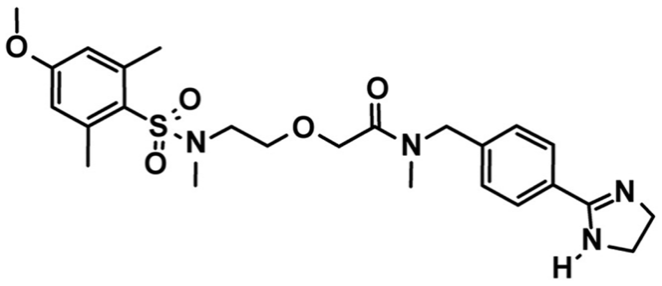
Chemical structure of LF22-0542.

Seven days after diabetes induction, rats were treated twice a day (8:30 AM and 5:30 PM) with one eye drop application of the water soluble B_1_R antagonist LF22-0542 (1% in saline) for a 7-day period. LF22-0542 (*N*-[[4-(4,5-dihydro-1*H*-imidazol-2-yl)phenyl]methyl]-2-[2-[[(4-methoxy-2,6 dimethylphenyl)sulfonyl]methylamino]ethoxy]-*N*-methyl-acetamide, fumarate) is a competitive non-peptide B_1_R antagonist which was synthesized and provided by Fovea Pharmaceuticals SA, Paris, France. The chemical structure of LF22-0542 is presented in Figure 1. LF22-0542 showed high affinity for human and mouse B_1_R with virtually no affinity for the human B_2_R; a selectivity index of at least 4000 times was obtained when LF22-0542 was profiled throughout binding or cell biology assays on 64 other G-protein-coupled receptor, 10 ion channels, and seven enzymes [25]. LF22-0542 blocked pain behavior in various inflammatory and neuropathic pain models in rats and mice and was found inactive in B_1_R knockout mice models of inflammatory pain [25], [26], [27]. Fresh solution was prepared daily by dissolving the compound in saline 0.9% and sterilizing the solution by filtration (0.20 µm mesh). For ocular instillation, rats were firmly maintained and a drop of solution (10 µl) was instilled on the surface of the eye using a micropipette. Animals were maintained 10 s in order to make sure that the drop effectively remained on the surface of the eye. Every day, the rats were visually inspected to detect the presence of ocular irritation such as redness, porphyrin secretion or corneal opacity. All end points were determined on the last day of treatment.

### Measurement of retinal vascular permeability

Retinal vascular permeability was assessed using Evans blue dye extravasation technique as previously described [17]. Rats were anesthetized with sodium pentobarbital (60 mg/kg, i.p.) and a catheter (Micro-Renathane, I.D. 0.040″, O.D. 0.020″, Braintree Scientific, Braintree, MA, USA) was inserted into the right femoral vein. Evans blue dye (45 mg/ml in saline) (Sigma-Aldrich, Oakville, ON, Canada) was injected i.v. over 10 s. The dye was allowed to circulate for 2 h and then saline (25 ml) was infused through the left ventricle, to wash out intravascular dye. Following enucleation, retinae were dissected out and immediately weighed. Evans blue dye was then extracted by incubating each retina in 1 ml formamide (Sigma-Aldrich, Oakville, ON, Canada) for 18 h at 70–75°C. The fluorescence of Evans blue was measured using a spectrofluorometer (Spex 1681 0.22 m, Horiba JobinYvon Inc, Edison, NJ, USA) at 620 nm (excitation) and 680 nm (emission).

### Measurement of retinal leukostasis

Rats were anesthetized with isoflurane, the chest cavity was opened and a 16G cannula was inserted into the left heart ventricle. Rats were perfused with phosphate buffer saline (PBS) over 1 min (35 ml/min) to eliminate erythrocytes and non-adherent leukocytes. Fluorescein-isothiocyanate(FITC)-coupled Concanavalin A lectin (20 µg/ml in PBS, 5 mg/kg; Vector Labs, Burlington, ON, Canada) was infused at 30 ml/min to label adherent leukocytes and vascular endothelial cells. Rats were perfused with 4% paraformaldehyde over 4 min followed by 1% Albumin in PBS over 1 min and PBS over 2 min (35 ml/min). Retinae were dissected out, flat mounted on a glass slide and imaged using a fluorescence microscope (Leica microsystems Co., Germany). The total number of leukocytes in each retina was determined under microscope examination at 40×.

### Measurement of retinal inflammatory mediators by quantitative RT-PCR

Rats were anaesthetized with sodium pentobarbital (60 mg/kg, i.p.) and the eyes were dissected out. The retinae were isolated and put in RNA*later* stabilization reagent (QIAGEN, Valencia, CA, USA). Total RNA was extracted from retinae using a commercial kit (QIAGEN, Valencia, CA, USA). First-strand cDNA synthesized from 400 ng total RNA with random hexamer primers was used as template for each reaction with the QuantiTect Rev Transcription Kit (QIAGEN). SYBR Green-based real-time quantitative PCR using Mx3000p device for signal detection (Stratagene, La Jolla, CA, USA) was performed as previously described [17]. PCR was performed in SYBR Green Master mix (QIAGEN) with 300 nM of each primer. The primer pairs designed by Vector NTI software are shown in Table 1. For standardization and quantification, rat 18S was amplified simultaneously. PCR conditions were as follows: 95°C for 15 min, followed by 46 cycles at 94°C for 15 s, 60°C for 30 s and 72°C for 30 s. The cycle threshold (Ct) value represents the cycle number at which a fluorescent signal rises statistically above background. The relative quantification of gene expression was analyzed by the 2^−ΔΔCt^ method [28].

**Table 1:** Primers list.

		Sequence			Position			Gen Bank
B_1_R	Forward	5′	GCA GCG CTT AAC CAT AGC GGA AAT	3′	367	-	390	NM_030851
	Reverse	5′	CCA GTT GAA ACG GTT CCC GAT GTT	3′	454	-	431	
B_2_R	Forward	5′	AGG TGC TGA GGA ACA ACG AGA TGA	3′	882	-	905	NM_173100
	Reverse	5′	TCC AGG AAG GTG CTG ATC TGG AAA	3′	990	-	967	
iNOS	Forward	5′	TGA TCT TGT GCT GGA GGT GAC CAT	3′	1150	-	1173	NM_012611
	Reverse	5′	TGT AGC GCT GTG TGT CAC AGA AGT	3′	1349	-	1326	
eNOS	Forward	5′	TAT TTG ATG CTC GGG ACT GCA GGA	3′	587	-	610	NM_021838
	Reverse	5′	ACG AAG ATT GCC TCG GTT TGT TGC	3′	678	-	655	
COX-2	Forward	5′	GCA TTC TTT GCC CAG CAC TTC ACT	3′	677	-	700	U03389
	Reverse	5′	TTT AAG TCC ACT CCA TGG CCC AGT	3′	744	-	751	
ICAM-1	Forward	5′	TGC AGG TGA ACT GCT CTT CCT CTT	3′	161	-	184	NM_012967
	Reverse	5′	AGC TTC CAG TTG TGT CCA CTC GAT	3′	263	-	240	
VEGF-A	Forward	5′	TCA CCA AAG CCA GCA CAT AGG AGA	3′	1219	-	1242	BC168708
	Reverse	5′	TTA CAC GTC TGC GGA TCT TGG ACA	3′	1371	-	1348	
VEGF-R_2_	Forward	5′	AGT GGC TAA GGG CAT GGA GTT CTT	3′	3269	-	3292	U93306
	Reverse	5′	GGG CCA AGC CAA AGT CAC AGA TTT	3′	3387	-	3364	
IL-1β	Forward	5′	TGT CAC TCA TTG TGG CTG TGG AGA	3′	247	-	270	NM_031512
	Reverse	5′	TGG GAA CAT CAC ACA CTA GCA GGT	3′	411	-	388	
HIF-1α	Forward	5′	TAG ACT TGG AAA TGC TGG CTC CCT	3′	1693	-	1716	NM_024359
	Reverse	5′	TGG CAGTGA CAG TGA TGG TAG GTT	3′	1863	-	1840	
18S	Forward	5′	TCA ACT TTC GAT GGT AGT CGC CGT	3′	363	-	385	X01117
	Reverse	5′	TCC TTG GAT GTG GTA GCC GTT TCT	3′	470	-	447	

### Measurement of superoxide anion production in the retina

Rats were sacrificed with CO_2_ inhalation and the eyes were dissected out, frozen in isopentane (−55°C), cut into 20-µm thick sections and placed on glass slides. Superoxide anion (O_2_^•−^) production was measured in retina using the oxidative fluorescent dye dihydroethidium (DHE) as described earlier [29]. Cells are permeable to hydroethidine and, in the presence of O_2_^•−^, it is oxidized to fluorescent ethidium bromide (EtBr) which is trapped by intercalation with DNA. EtBr is excited at 518 nm with an emission spectrum of 605 nm. Dihydroethidium (2 µM) (Sigma-Aldrich, Oakville, ON, Canada) was applied to 20-µm thick eye sections and the slides were then incubated in a light-protected humidified chamber at 37°C for 30 min. Nuclei of retinal cells were stained with TO-PRO-3 (Molecular Probes, Eugene, Ore, USA). Images were obtained with a Leica TCS SP confocal microscope equipped with an argon laser (Leica microsystems Co., Germany). Tissues from each experimental group were processed and imaged in parallel. Laser settings were identical for acquisition of images from all sections. Leica LCS Lite software was used to quantify the mean pixel energy of DHE and TO-PRO-3 in the retinal ganglion cells (RGC) layer, the inner nuclear layer (INL) and the outer nuclear layer (ONL). Data were expressed as the mean pixel energy ratio between DHE and TO-PRO-3 from an average of 10 nuclei in the RGC layer or 20 nuclei in the INL and ONL for each retinal image quantified in 3 rats.

### Statistical analysis

Data were expressed as mean ± s.e.m. and *n* represents the number of rats used in each experiment. Multiple comparisons between groups were performed using the non-parametric Mann-Whitney test for retinal leukostasis. One-way ANOVA and the Bonferroni *post-hoc* test were used for Table 2 data, vascular permeability, expression of inflammatory mediators and superoxide anion measurement. Only probability values (P) less than 0.05 were considered to be statistically significant.

**Table 2:** Effect of diabetes and LF22-0542 on glycemia and body weight.

	Glycemia (mmol/L)			Body weight (g)		
Control+Vehicle (n = 7)	5.5	±	0.2	356	±	6
Control+LF22-0542 (n = 6)	5.1	±	0.2	355	±	5
STZ+Vehicle (n = 5 )	26.8	±	2.9***	295	±	9***
STZ+LF22-0542 (n = 7)	27.7	±	2.9***	274	±	5***

Values are mean ± s.e.m.

*** P<0.001, significantly different from control group.

## Results

### Physiological parameters

As shown in Table 2, blood glucose concentration was significantly increased in STZ-diabetic rats at the time of sacrifice when compared to age-matched control rats (P<0.001). One-week eye drops application of LF22-0542 had no effect on glycemia in both control and STZ-diabetic rats (P>0.05). Body weight was significantly reduced in STZ-diabetic rats treated or not with LF22-0542 compared to controls (P<0.001). Treatment of diabetic rats with LF22-0542 had no effect on body weight when compared to diabetic rats treated with the vehicle (P>0.05). Control and STZ-diabetic rats that received eye drops of LF22-0542 did not show symptoms of ocular irritation (redness or corneal opacity) or the presence of porphyrin secretion around the eyes during the whole period of treatment.

### Effect of LF22-0542 on retinal vascular permeability

**Figure 2:**
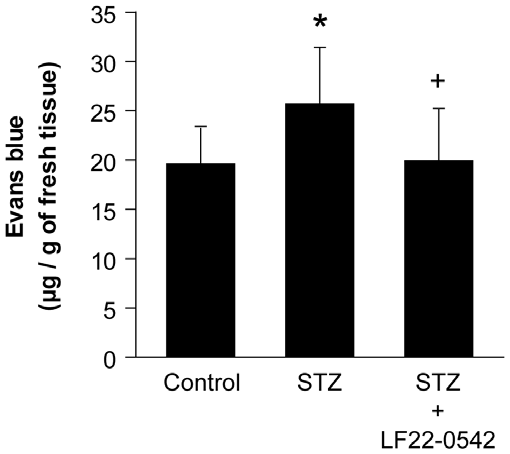
Effect of LF22-0542 on retinal vascular permeability in STZ-diabetic rats. Data are mean ± s.e.m. of values obtained from 9 to 11 rats. Statistical comparison with control (*) or STZ (+) rats is indicated by *^+^P<0.05.

Evans Blue extravasation (µg/g of fresh tissue) was significantly increased by 31% in STZ-diabetic rats compared to control rats (P<0.05). Topical ocular administration of LF22-0542 reversed retinal vascular hyperpermeability to control values in STZ-diabetic rats (P<0.05) (Figure 2).

### Effect of LF22-0542 on retinal leukostasis

**Figure 3:**
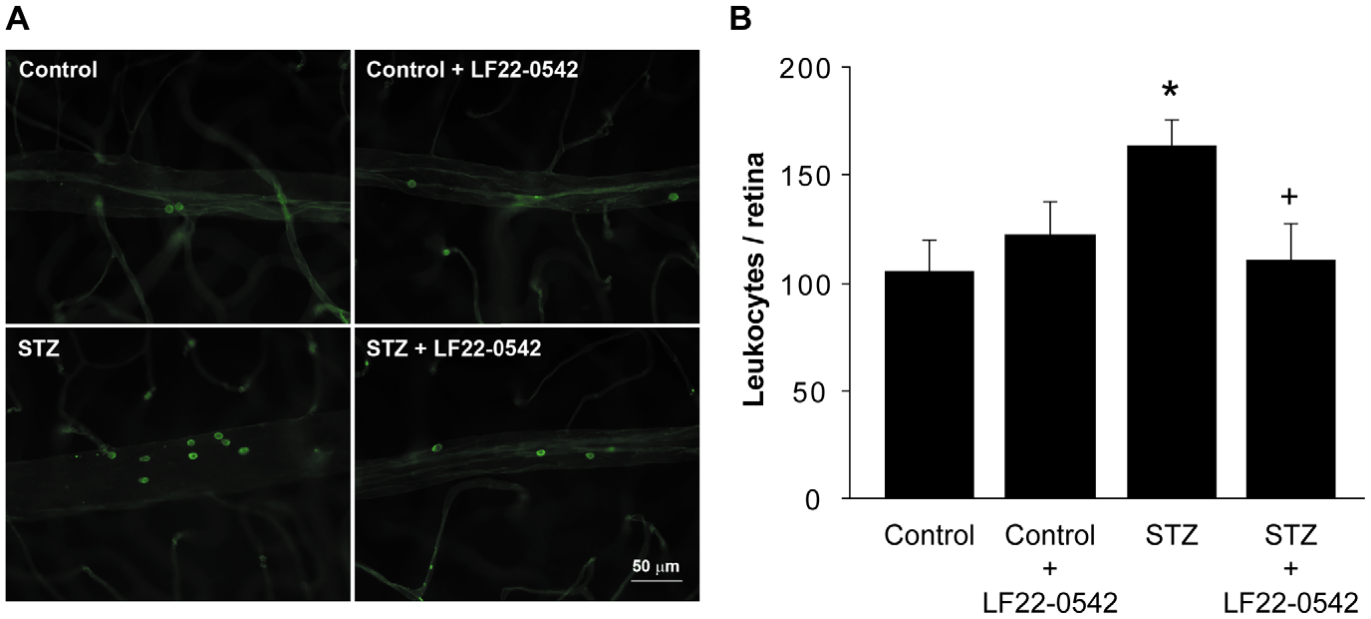
Effect of LF22-0542 on retinal leukostasis in STZ-diabetic rats. (A) Representative pictures of adherent leucocytes in retinal vessels of a control rat, a control rat treated with LF22-0542, a STZ-diabetic rat and a STZ-diabetic rat treated with LF22-0542. Scale bar is 50 µm. (B) Number of adherent leukocytes per retina. Data are mean ± s.e.m. of values obtained from 5 to 7 rats in each group. Statistical comparison with control (*) or STZ (+) rats is indicated by *^+^P<0.05.

Retinal adherent leukocytes were labelled with FITC-Concanavalin A lectin (Figure 3A). Total number of adherent leukocytes in the retinal wall was significantly increased in STZ-diabetic rats compared to control rats (P<0.05) (Figure 3B). One-week eye drops administration of LF22-0542 to diabetic rats significantly decreased retinal leukostasis (P<0.05). B_1_R antagonist treatment had no significant effect on leukocytes number in control retina (P>0.05).

### Effect of LF22-0542 on the expression of inflammatory mediators

**Figure 4:**
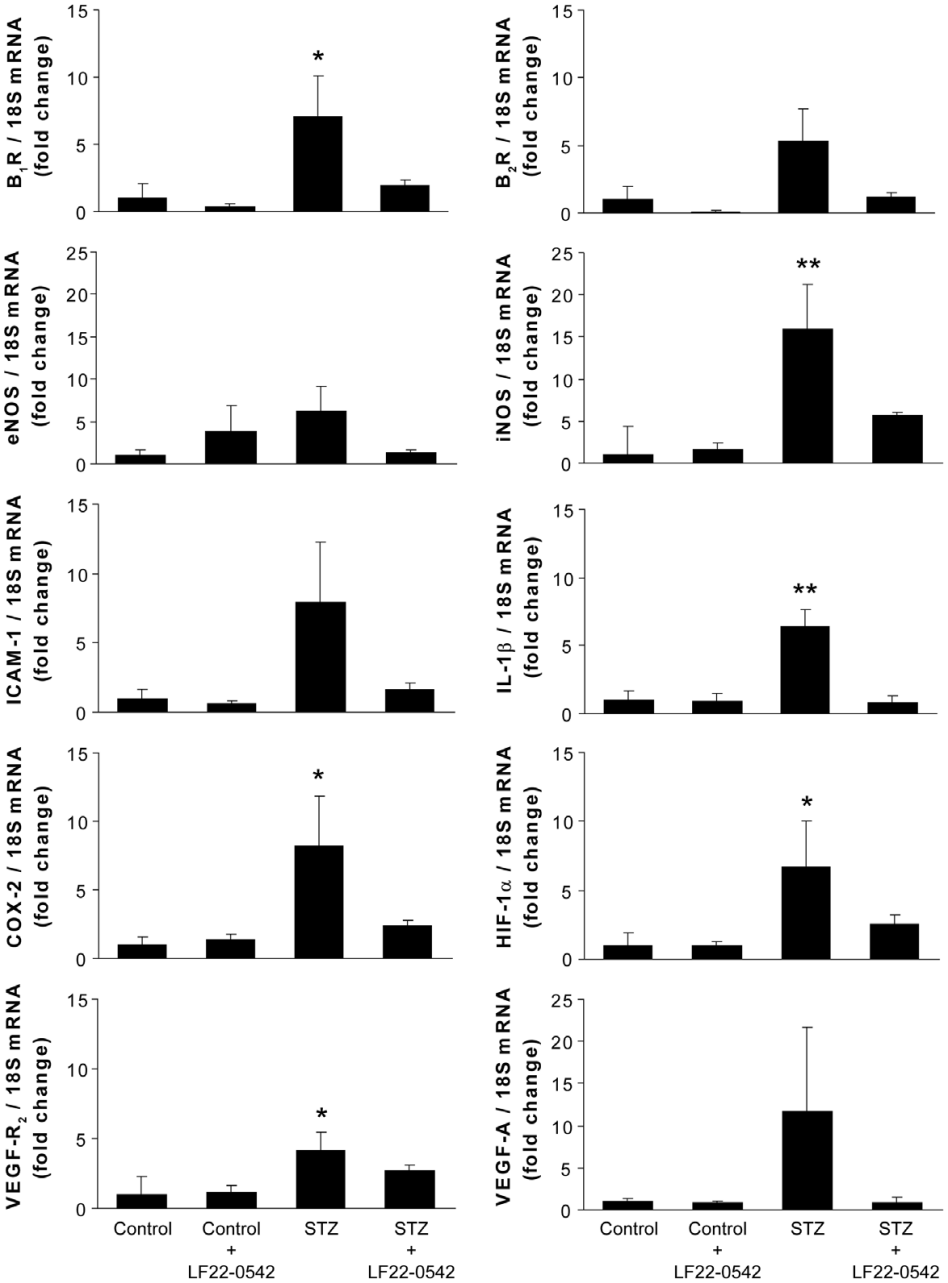
Effect of LF22-0542 on the expression of retinal inflammatory mediators in STZ-diabetic rats. mRNA levels of B_1_R, B_2_R, eNOS, iNOS, ICAM-1, IL-1β, COX-2, HIF-1α,VEGF-R_2_ and VEGF-A. Data are mean ± s.e.m. of values obtained from 7–8 rats in each group. Statistical comparison with control rats (*) is indicated by *P<0.05, **P<0.01.

All inflammatory mediators were expressed at very low levels in the retina of control rats (Figure 4). In STZ-diabetic rats (*n* = 7), retinal mRNA levels of B_1_R, iNOS, IL-1β, COX-2, VEGF-R_2_ and HIF-1α were significantly increased (4 to 16-fold) compared to control rats (*n* = 7, P<0.05). These increases were restored to control values in rats treated with LF22-0542 (*n* = 7). The increase mRNA levels of B_2_R, ICAM-1, eNOS and VEGF-A did not reach statistical significance in STZ-diabetic retinae, yet LF22-0542 treatment abolished to control values this trend.

### Effect of LF22-0542 on oxidative stress

**Figure 5:**
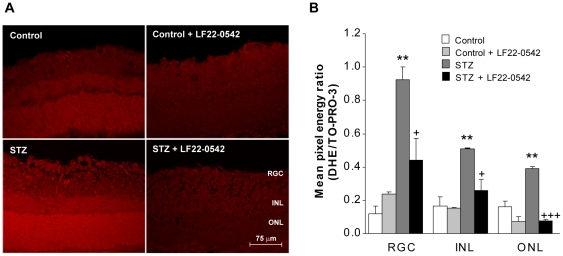
Effect of LF22-0542 on retinal oxidative stress in STZ-diabetic rats. (A) Representative pictures of superoxide anion production stained with dihydroethidine on retinal section from a control rat, a control rat treated with LF22-0542, a STZ-diabetic rat and a STZ-diabetic rat treated with LF22-0542. Scale bar is 75 µm. (B) Fluorescence intensity of superoxide anion was quantify by the evaluation of mean pixel energy ratio of DHE staining versus TO-PRO-3 in the retinal ganglion cells layer (RGC), the inner nuclear layer (INL) and the outer nuclear layer (ONL). Data are mean ± s.e.m. of values obtained from 3 rats in each group. Statistical comparison with control (*) or STZ (+) rats is indicated by **P<0.01, ^+^P<0.05, ^+++^P<0.001.

Production of superoxide anion (O_2_^•−^) in the retina measured with the oxidative fluorescent dye dihydroethidine is shown in Figure 5. DHE staining of STZ-diabetic retinal sections displayed a higher intensity of fluorescence than controls (Figure 5A). Quantification of the O_2_^•−^ retinal levels (mean pixel energy ratio of DHE staining versus TO-PRO-3) showed a significant increase in the retinal ganglion cells layer (RGC) (P<0.01), the inner nuclear layer (INL) (P<0.01) and the outer nuclear layer (ONL) (P<0.01) of STZ-diabetic rats compared to control rats (Figure 5B). Importantly, one-week topical administration of LF22-0542 to STZ-diabetic rats markedly reduced O_2_^•−^ levels in the 3 retinal nuclear layers (P<0.05). Administration of LF22-0542 had no significant effect on basal production of O_2_^•−^ in the retina of control animals (P>0.05).

## Discussion

This study provides the first demonstration that prolonged ocular application of a water soluble non-peptide kinin B_1_R antagonist is an effective and non-toxic approach to inhibit diabetes-induced retinal inflammation and oxidative stress. The results clearly show that the B_1_R antagonist reversed the increased vascular permeability, leukostasis, the enhanced expression of several inflammatory mediators and the higher production of reactive oxygen species.

### Retinal vascular permeability

Breakdown of the blood-retinal barrier appears early in the progression of diabetic retinopathy and often leads to the development of macular edema which is a major cause of visual impairment in diabetic patients [30], [31]. Retinal vascular permeability has been largely studied in experimental models of diabetes and evidence was provided that breakdown of the blood-retinal barrier occurs as early as one week following diabetes induction in rats [24], [32]. The present study shows that 7-day eye drops application of the B_1_R antagonist LF22-0542 completely reversed increased retinal vascular permeability in 2-week STZ-diabetic rats. This is consistent with the inhibition of retinal plasma extravasation following a 7-day i.v. treatment with the peptide B_1_R antagonist R-715 in STZ-diabetic rats [17]. Likewise, s.c. or i.v. injection of the peptide B_1_R antagonist R-954 reversed the enhanced vascular permeability in the retina and other target tissues in mice and rats treated with STZ 1 and 4 weeks earlier [33], [34], [35]. Our results are also consistent with a previous study that showed a decrease in retinal vascular permeability following treatment with a selective plasma kallikrein inhibitor in STZ-diabetic rats [36].

The alleviation of enhanced vascular permeability by B_1_R antagonism could be explained by the concomitant reduction in gene expression of iNOS, COX-2 and VEGF-R_2_ in rats treated with LF22-0542. Nitric oxide pathway, prostaglandins and VEGF are known to play a role in breakdown of the blood-retinal barrier through a mechanism involving the upregulation of intercellular adhesion molecules (ICAM-1) and the downregulation of tight junctions proteins in the retinal vasculature [37], [38], [39]. Moreover, B_1_R is known to be expressed on retinal vessels and could therefore directly increase vascular permeability through the release of NO and prostaglandins following its activation.

In addition to supporting a key role for kinin B_1_R in retinal vascular permeability, our results demonstrate that LF22-0542 reached the rat retina when given by eye drops application. This is the first use of a B_1_R antagonist as ocular treatment. LF22-0542 had a specific action at the ocular level and was not detected in the blood circulation after prolonged ocular administration in rabbits (unpublished data). Therefore, eye drops application provides the advantage of avoiding systemic effects for potential clinical treatment. Moreover, topical treatment is the less invasive route of administration for treatment of ocular disease and would be beneficial for the treatment of diabetic retinopathy. Current therapies for these patients include laser induced retinal photocoagulation and repetitive intravitreous injections of anti-VEGF molecules, which are very expensive and associated with significant risks of infection, retinal lesion and pain. These treatments help to reduce the progression of diabetic retinopathy in advanced stages of the disease but are not curative. Topical B_1_R antagonist is therefore a very promising approach for the treatment of early pathological changes in the diabetic retina.

### Leukostasis

Leukocytes adhesion to the retinal vasculature is thought to be associated with endothelial cell death, capillary occlusion and increased vascular permeability, which all contribute to the progression of diabetic retinopathy [21], [22]. Following their adherence to the retinal vessels of diabetic rats, leukocytes exit the vasculature and transmigrate to the neural retina [24], [40], [41]. Our data suggest that B_1_R antagonism is a possible therapeutic strategy for reversing retinal leukostasis and the associated vascular alterations in diabetes. Previously, B_1_R has been shown to be involved in all 3 phases of the leukocyte recruitment process in inflamed tissue [42], [43]. B_1_R agonist caused leukocytes rolling, adhesion and emigration in mouse mesenteric postcapillary venules [44] and leukocytes migration and infiltration in the pleural cavity [45], [46].

Leukocytes bind to ICAM on the surface of endothelial cells to adhere to the vasculature. Previous studies reported that the increased number of adherent leukocytes in the diabetic retina is mediated by the concomitant increase of ICAM-1 expression in the retinal vasculature [24], [47]. Importantly, our results show a reduced level of expression of ICAM-1 in the retina of STZ-diabetic rats treated with LF22-0542 in parallel with the decrease of leukostasis.

### Expression of B_1_R and B_2_R

Real time RT-PCR analysis revealed that B_1_R was significantly upregulated in the retina of STZ-diabetic rats which is consistent with the increased density of B_1_R binding sites previously reported by autoradiography [16] and with the increased B_1_R protein expression by western blot [48]. The activation of B_1_R is known to induce the production of pro-inflammatory cytokines that can directly cause the expression of B_1_R. The prolonged ocular treatment with B_1_R antagonist inhibits both the inflammatory response and expression of B_1_R. Level of B_2_R gene expression in the retina of diabetic rats was slightly but not significantly increased in the diabetic retina, yet it was reduced to control values in diabetic rats treated with LF22-0542. Further studies are needed to clarify the contribution of B_2_R in the effects mediated by B_1_R blockade.

### Expression of inflammatory mediators

Our results demonstrate that B_1_R plays a central role in retinal inflammation by interacting with many pro-inflammatory factors involved in vascular alterations. mRNA levels of IL-1β, COX-2 and VEGF-R_2_ were increased in diabetic retinas compared to control rats and were restored to control values with LF22-0542 treatment. Previous studies also reported increased expression of IL-1β and COX-2 in the retina of diabetic rats [49], [50]. VEGF is also known to play a central role in the development of diabetic retinopathy since it is involved in neovascularisation and increased vascular permeability [38], [39], [50]. Moreover, our results show that iNOS gene expression was significantly increased in the diabetic retina as previously shown in diabetic rodents and patients [51], [52], and this was normalized by LF22-0542. Whereas B_2_R-mediated eNOS activation leads to a transient (5 min) output of NO, B_1_R-mediated iNOS activation leads to a very high and prolonged (90 min) NO production in human endothelial cells which is consistent with the detrimental effect of iNOS on vascular function [53]. The protective vascular function of eNOS through the release of NO appears intact in STZ-diabetic retina at this early stage as evidenced by the lack of alterations in eNOS expression. This is supported by a recent study on retinal blood flow showing that the endothelium-dependent relaxation mediated by B_1_R agonist was not impaired in this model up to 6 weeks post-STZ (Pouliot, M., Hétu S.,Vaucher, E., Couture, R., unpublished data).

Our results also demonstrate that HIF-1α was increased in diabetic retina and normalized with B_1_R antagonist. HIF-1α is a transcription factor that accumulates in cells during hypoxia and heterodimerizes with the constitutively expressed HIF-1β subunit, triggering the activation of many genes such as VEGF. Retinal HIF-1α was previously found to be activated in 2-week STZ-diabetic rats and to directly regulate VEGF gene expression [54]. Ischemia takes place early in the diabetic retina and our results show that B_1_R antagonism may be effective in reducing HIF-1α expression and subsequent retinal ischemia.

### Oxidative stress

Compelling evidence suggests that oxidative stress induced by hyperglycemia plays an important role in the development of vascular alterations in the retina [19], [55]. Oxidative stress is produced by multiple cell types in the retina including endothelial cells, pericytes and glial cells [19], [56], [57], [58]. In agreement with previous studies conducted in diabetic mice and rats [56], [59], [60], we found increased production of superoxide anion in the retina of STZ-diabetic rats, which was reversed by LF22-0542. Since retinal B_1_R overexpression was reversed by an antioxidant in STZ-diabetic rats [17], it was concluded that the oxidative stress associated with hyperglycemia is responsible for the induction of B_1_R in the retina. Herein, data suggest that the stimulation of B_1_R can perpetuate the harmful production of ROS, and therefore the blockade of B_1_R should interrupt this deleterious vicious cycle, since inflammation can induce oxidative stress and *vice versa*. This is in keeping with other relevant findings. In cardiac tissue, nitrotyrosine protein level, a marker of oxidative stress, was reduced in B_1_R knockout diabetic mice [61]. In a rat model of insulin resistance, one-week treatment with the orally active B_1_R antagonist SSR240612 reversed increased levels of superoxide anion and B_1_R expression in the aorta through the inhibition of NADPH oxidase [62].

The alleviation of oxidative stress in the diabetic retina by B_1_R antagonist could explain the reduction in the expression of inflammatory mediators. It is known that oxidative stress resulting from the production of ROS triggers the activation of the nuclear transcription factor kappa B (NF-κB) [63]. NF-*κ*B is a widely expressed inducible transcription factor and an important regulator of many genes involved in inflammatory and immune responses including IL-1β, ICAM-1, iNOS, COX-2 and B_1_R [20], [64]. Therefore, by reducing oxidative stress in the retina, B_1_R antagonist could indirectly inhibit NF-*κ*B activation and thereby inflammation, including the expression of its own receptor.

### Conclusion

The findings suggest that B_1_R is involved in the inflammatory cascade leading to retinal vascular damage in the early stages of diabetes. The oxidative stress associated with hyperglycemia induces the expression of B_1_R in the retina. In turn, the activation of B_1_R may perpetuate the production of ROS to enhance the expression of vascular pro-inflammatory mediators (COX-2, IL-1β, ICAM-1, VEGF and HIF-1α). Those mediators in concert with B_1_R could enhance leukostasis and vascular permeability in the diabetic retina. These pathological events are likely to contribute to the development of diabetic retinopathy. Hence, the ocular application of LF22-0542, a highly potent antagonist at human B_1_R, represents a promising therapeutic approach in diabetic retinopathy.
